# Assessment of Concurrent Chemoradiotherapy plus Induction Chemotherapy in Advanced Nasopharyngeal Carcinoma: Cisplatin, Fluorouracil, and Docetaxel versus Gemcitabine and Cisplatin

**DOI:** 10.1038/s41598-018-33614-5

**Published:** 2018-10-22

**Authors:** Zhen Zeng, Ruo-Nan Yan, Li Tu, Yu-Yi Wang, Pei-Ran Chen, Feng Luo, Lei Liu

**Affiliations:** 1 Department of Medical Oncology, Cancer Center, State Key Laboratory of Biotherapy, and Lung Cancer Center, West China Hospital of Sichuan University, Chengdu, 610041 Sichuan China; 2 Department of Head and Neck Oncology, Cancer Center, and State Key Laboratory of Biotherapy, West China Hospital of Sichuan University, Chengdu, 610041 Sichuan China; 3 Sichuan University, Chengdu, 610041 Sichuan China

## Abstract

Induction chemotherapy treatment for nasopharyngeal carcinoma (NPC) is controversial. The aim of this study was to evaluate the treatment outcomes and toxicities between two induction chemotherapy regimens, with both followed by concurrent chemoradiotherapy. The first strategy used docetaxel, cisplatin and fluorouracil for induction chemotherapy (TPF), and the second utilised gemcitabine and cisplatin (GP). A retrospective analysis was performed on eligible NPC patients attending our hospital between May 2009 and Dec 2014. A total of 113 patients were enrolled with 58 patients receiving TPF and 55 receiving GP induction chemotherapy. Ninety-four patients (83.2%) were alive after 36-months follow-up. The median overall survival (OS) and progression-free survival (PFS) time were 48.3 and 39.7 months, respectively. The 3-year OS for the TPF regimen was 87.9% and 87.4% with GP chemotherapy (P = 0.928). The 3-year PFS of the TPF treatment was 84.5%, while it was 83.5% for the GP group (P = 0.551). Univariate analysis showed that lymph node metastasis was a significant PFS prognostic factor, while N3 stage was an independent predictor of PFS and distant failure-free survival (DMFS) in multivariate analysis. There were no significant differences in adverse toxicities or treatment efficacy between the chemotherapy regimens in the treatment of locoregionally advanced NPC.

## Introduction

In 2012, there were nearly 86,700 new cases and 50,800 deaths resulting from nasopharyngeal carcinoma (NPC), which is a particularly prevalent carcinoma in a select geographic and ethnic population. Among the Chinese population, NPC shows a high incidence, especially in South-Eastern China, including the Guangdong province and the Hong Kong area^1^. NPC is sensitive to radiation and radiotherapy (RT), and RT is one of the primary treatment strategies for NPC^2^ because of anatomical constraints. In addition, chemotherapy is also used to treat NPC^3^. Substantial evidence indicates that a combination of RT and chemotherapy, known as chemoradiotherapy, leads to better outcomes in comparison to RT alone for patients with advanced NPC^4^.

The NPC-9901 clinical trial assessed the therapeutic benefits of chemoradiotherapy, and the findings have laid the foundation for concurrent chemoradiotherapy (CCRT) to become the standard treatment for locoregionally advanced NPC^5,6^. With the advancement of medicine and modern delivery technologies for radiation treatment, intensity-modulated radiotherapy (IMRT) has changed the prognosis for NPC. There are some patients showing complete responses with RT^7^. However, the 5-year survival rate for RT alone is limited^8^. Distant metastasis is a difficult problem for NPC patients^9^, indicating a requirement for systemic therapy. Chemotherapy as a type of systemic therapy has been suggested for NPC. Adding chemotherapy to the treatment of locally advanced NPC has been shown to benefit patients^10^.

Recently, a phase 3 randomised controlled trial comparing CCRT alone versus induction chemotherapy including docetaxel, cisplatin and fluorouracil (5-FU) (known as TPF) followed by CCRT, demonstrated significant improvements in failure-free survival for patients with locoregionally advanced NPC. However, the TPF induction regimen had more side-effects than CCRT alone especially in relation to neutropenia and leukopenia, with significantly higher proportions of grade 3–4^6^. Kawahira *et al*. revealed that induction TPF might reduce distant metastasis^11^. Another study reported that gemcitabine and cisplatin (GP) induction chemotherapy demonstrated better overall survival (OS), and improved distant metastasis-free survival (DMFS) to some extent^12^. Further, research has confirmed that the GP induction regimen conferred a survival benefit in recurrent or metastatic NPC^13^. Wang *et al*. found that GP-based induction chemotherapy was effective with acceptable toxicities^14^. In addition, a phase II study also concluded that GP induction chemotherapy with IMRT, was well-tolerated and effective for locoregionally advanced NPC^15^.

Consequently, in the present study we aim to compare the TPF induction regimen (cisplatin, 5-FU, and docetaxel) with the GP regimen (gemcitabine and cisplatin) in patients with locoregionally advanced NPC, with both treatment groups also receiving CCRT.

## Results

There were 113 patients in total in our study, aged between 22.0 and 66.9 years. The average age for all patients was 47.0 years. In the two matched groups, there was no significant difference in age, gender, tumour staging and Eastern Cooperative Oncology Group (ECOG) scoring. The average age in the TPF and GP groups were 45.9 (22–66) and 48.2 (26–65) years, respectively (P = 0.357). All patients accepted induction chemotherapy plus CCRT. Patients with previously untreated NPC (except distant metastasis) were fitted into our final analyses. The average follow-up time was 51.4 (16.8–98.3) months. Baseline characteristics are summarised in Table 1. 47 (81%) patients completed three cycles of TPF induction chemotherapy, 11 (19%) received two cycles. In GP group, 47 (85.5%) patients accepted three cycles of induction chemotherapy and 8 (14.5%) completed two cycles. During the time of CCRT, 83.2% of patients completed three cycles and 10.6% had two cycles. Only one patient postponed CCRT because of adverse events. Table 2 shows the detail compliance of IC and concurrent chemotherapy between two arms.

**Table 1:** Baseline characteristics of the 113 patients receiving TPF and GP treatment regimens.

Characteristic	TPF regimen n = 58	GP regimen n = 55	n = 113	p value
gender				0.207
male	47 (81.0%)	39 (70.9%)	86 (76.1%)	
female	11 (19.0%)	16 (29.1%)	27 (23.9%)	
average age,years	45.9	48.2	47	0.357
T category				0.306
T1	7 (12.1%)	10 (18.2%)	17 (15.0%)	
T2	7 (12.1%)	6 (11.3%)	13 (11.5%)	
T3	19 (33.7%)	10 (18.2%)	29 (25.7%)	
T4	25 (43.1%)	29 (52.3%)	54 (47.8%)	
N category				0.611
N0	3 (5.2%)	5 (9.1%)	8 (7.1%)	
N1	14 (24.1%)	10 (18.2%)	24 (21.2%)	
N2	33 (56.9%)	29 (52.7%)	62 (54.9%)	
N3	8 (13.8%)	11 (20.0%)	19 (16.8%)	
Disease stage				0.574
III	23 (39.7)	19 (34.5%)	42 (37.2%)	
IV	35 (60.3%)	36 (65.5%)	71 (62.8%)

**Table 2:** The detail compliance of IC and concurrent chemotherapy between two arms.

	TPF regimen	GP regimen	P value
**Induction chemotherapy**			
Total No. of cycles given, %			0.53
Two cycles	11 (19.0%)	8 (14.5%)	
Three cycles	47 (81.0%)	47 (85.5%)	
**Concurrent chemotherapy**			
Total No. of cycles given, %			0.82
One cycles	4 (6.9%)	3 (5.4%)	
Two cycles	7 (12.1%)	5 (9.1%)	
Three cycles	47 (81.0%)	47 (85.5%)	

**Figure 1:**
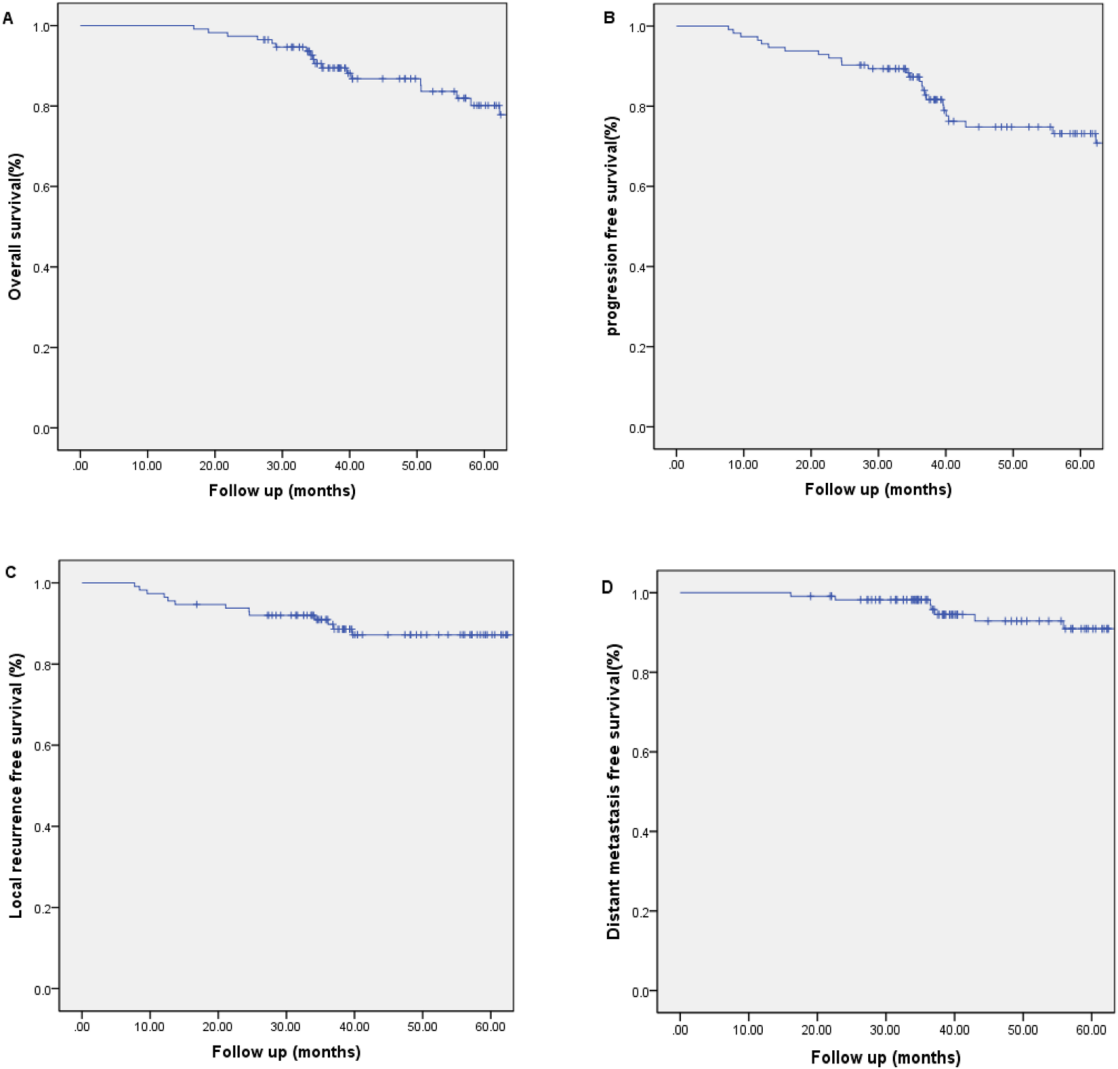
Overall survival, progression-free survival, local recurrence-free survival and distant metastasis-free survival rates of induction chemotherapy-concurrent chemoradiotherapy (IC-CCRT) in NPC patients.

In all, a total of 94 patients (83.2%) were alive at the end of the study. The survival outcomes for the entire patient cohort were shown in Fig. 1. The 3- and 5-year estimated OS, PFS, LRFS and DMFS were 87.9%, 84.0%, 96.4%, and 87.5%, and 79.8%, 72.4%, 89.0% and 82.3%, respectively. The median OS time and PFS time were 48.3 months and 39.7 months.

**Figure 2:**
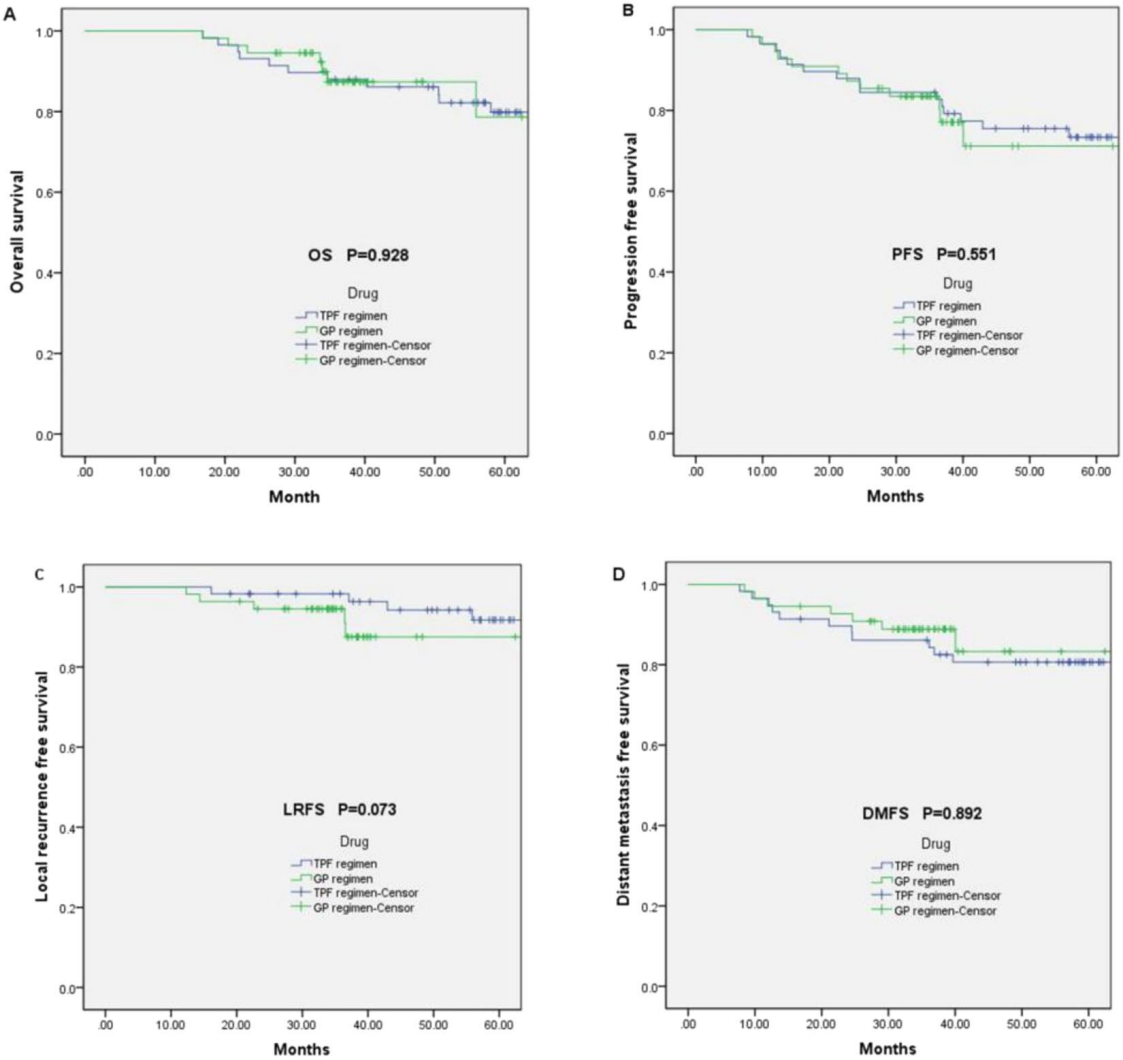
Kaplan–Meier survival curves. (**A**) Kaplan–Meier curve estimates for OS; (**B**) Kaplan–Meier curve estimates for PFS; (**C**) Kaplan–Meier curve estimates for LRFS; (**D**) Kaplan–Meier curve estimates for DMFS.

After induction chemotherapy, the efficacy in primary tumour and neck nodes was 92.9%, respectively. Short-term treatment responses were evaluated 3 months after radiation and the effectiveness of the primary tumour and neck nodes was 98.2% (the CR rate is 32.7%). Twelve (20.7%) patients’ deaths resulting from the NPC tumour(s) occurred in the TPF induction chemotherapy with CCRT group, compared to seven (12.7%) in the GP induction chemotherapy CCRT regimen. OS did not differ significantly between the two groups (Fig. 2A). The 3-year estimated OS of patients treated with TPF induction chemotherapy regimen with CCRT was 87.9% (95% CI 77.6–91.2) and it was 87.4% (95% CI 66.6–79.6) for the GP induction CCRT group (Log-Rank P = 0.928; Fig. 2A). The 3-year PFS for the TPF regimen was 84.5%, while it was 83.5% for the GP regimen (Log-Rank P = 0.551; Fig. 2B). No significant differences in PFS rates between the 2 arms were observed (Log-Rank P = 0.551; Fig. 2B).

Twenty-eight patients failed to respond to the treatments, including recurrence and metastasis, and death. The overall failure rate for all patients was 24.8%. Among them, 9 patients had recurrent disease and 19 patients presented with metastases. Pulmonary metastasis occurred 5 cases, liver metastasis occurred in 6 cases, bone metastasis occurred in 6 cases and pleural metastasis occurred in 1 case. The average time for local regional recurrence was 50.2 months. The average time for distant metastasis was 48.6 months. Patients in the TPF induction chemotherapy plus CCRT group, did not show better LRFS outcomes in comparison to those in the GP induction group (Log-Rank P = 0.073; Fig. 2C). Furthermore, DMFS rates were not different between the treatment groups (Log-Rank P = 0.892; Fig. 2D).

Univariate and multivariate regression analysis models assessed the potential prognostic factors associated with OS, PFS, LRFS and DMFS. We analysed gender, age, T stage, N stage, tumour stage, treatment group, haemoglobin reduction, leukopenia, thrombocytopenia, nausea and vomiting, diarrhoea, rash, hearing loss, alanine aminotransferase, aspartate aminotransferase, ECOG score as possible prognostic factors for patients included in this study.

N stage (lymph node metastasis) was found to be significantly associated with poorer PFS in a univariate analysis. Nausea and vomiting, ECOG performance, and N stage were independent prognostic factors predicting poorer DMFS rates. Rash onset predicated poorer outcomes in all follow-up end points. Specifically, high grade rash predicted poorer OS, PFS, LRFS and DMFS outcomes.

Following the univariate analyses, we chose treatment group (TPF regimen vs GP regimen), gender (male vs female), age (<50 vs >50), T stage, N stage, tumour stage and rash for the multivariate model. Treatment regimen was not found to be an independent prognostic factor for OS, PFS, LRFS and DMFS (Table 3). However, patients that developed a high grade rash (Table 3) following induction chemotherapy, were associated with significantly poorer prognostic outcomes for OS (HR = 2.916 95% CI = 1.388–6.125; P = 0.005), PFS (HR = 2.526 95% CI = 1.407–4.537; P = 0.002), LRFS (HR = 3.641 95% CI = 1.365–9.714; P = 0.010) and DMFS (HR = 2.307 95% CI = 1.141–4.664; P = 0.020). N3 was an independent prognostic element for PFS and DMFS. An analysis of covariates (e.g., N1 VS N2, N2 VS N3) for interaction with the treatment outcomes, demonstrated significant interaction (Table 3).

**Table 3:** Summary of multivariable analyses of prognostic factors.

	HR (95% CI)	p value
Overall survival		
treament group	1.422 (0.506–4.001)	0.504
Gender	4.017 (0.508–31.779)	0.188
Age (<50 vs >50)	2.083 (0.808–5.369)	0.129
T3 vs T1–2	1.343 (0.385–4.680)	0.643
T4 vs T1–2	0.688 (0.135–3.520)	0.654
N1 vs N3	0.368 (0.052–2.590)	0.316
N2 vs N3	0.337 (0.070–1.624)	0.175
grade	1.454 (0.248–8.528)	0.678
Rash	2.916 (1.388–6.125)	0.005
Progression-free survival		
treament group	1.248 (0.526–2.963)	0.615
Gender	1.189 (0.385–3.668)	0.764
Age (<50 vs >50)	1.601 (0.727–3.526)	0.243
T3 vs T1–2	0.694 (0.220–2.196)	0.535
T4 vs T1–2	1.406 (0.333–5.930)	0.643
N1 vs N3	0.301 (0.064–1.423)	0.130
N2 vs N3	0.258 (0.069–0.962)	0.044
grade	2.047 (0.396–10.595)	0.393
Rash	2.526 (1.407–4.537)	0.002
Local recurrence-free survival		
treament group	0.326 (0.075–1.418)	0.135
Gender	0.591 (0.105–3.319)	0.550
Age (<50 vs >50)	1.075 (0.249–4.642)	0.923
T3 vs T1–2	1.806 (0.277–11.780)	0.537
T4 vs T1–2	0.562 (0.048–6.518)	0.645
N1 vs N3	1.975 (0.148–26.302)	0.606
N2 vs N3	0.582 (0.070–4.848)	0.617
grade	0.491 (0.047–5.086)	0.551
Rash	3.641 (1.365–9.714)	0.010
Distant metastasis free survival		
treament group	2.248 (0.735–6.875)	0.156
Gender	1.487 (0.315–7.012)	0.616
Age (<50 vs >50)	1.836 (0.710–4.752)	0.210
T3 vs T1–2	0.343 (0.067–1.757)	0.199
T4 vs T1–2	2.204 (0.366–13.258)	0.388
N1 vs N3	0.119 (0.015–0.934)	0.043
N2 vs N3	0.175 (0.033–0.928)	0.041
grade	4.530 (0.505–40.667)	0.177
Rash	2.307 (1.141–4.664)	0.020

HR = hazard ratio.

Chi-square and/or Fisher’s exact tests were used to determine whether T stage, N stage, clinical stage and groups were independent, based on the treatment groups. The results showed no significant difference in T (P = 0.319), N (P = 0.088) and clinical stage (P = 0.558) distribution among two treatment groups. Subgroup analyses revealed that TPF compared with GP induction chemotherapy showed no significantly improved treatment outcomes, whether in OS or PFS, in patients with T3~4N1~3M0, Stage III tumours, or Stage IV tumours (Table 4).

**Table 4:** Subgroup analysis.

Subgroup analysis	p value TPF regimen VS GP regimen
T3~4N1~3M0	
Overall survival	0.875
Progression-free survival	0.543
stage III	
Overall survival	0.646
Progression-free survival	0.806
stage IV	
Overall survival	0.483
Progression-free survival	0.538

The toxicities of induction chemotherapy and radiotherapy by site and grade are summarised in Table 5. During induction chemotherapy, all patients completed the treatment without interruption and tolerated the toxicity. Only one patient was observed with grade IV toxicity and one patient postponed the CCRT because of high grade radiation-toxicity.

**Table 5:** Toxicities of induction chemotherapy and radiotherapy.

Toxicities	Toxicity Grade					P value
	0	1	2	3	4	TPF vs GP
The toxicities of IC						
Leukopenia	21 (18.6%)	21 (18.6%)	54 (47.8%)	17 (15.0%)	0 (0.0%)	0.189
Thrombocytopenia	78 (69.0%)	24 (21.2%)	7 (6.2%)	3 (2.7%)	1 (0.9%)	0.794
Hemoglobin reduction	63 (55.8%)	48 (42.5%)	2 (1.8%)	0 (0.0%)	0 (0.0%)	0.176
Nausea and vomiting	55 (48.7%)	49 (43.4%)	8 (7.1%)	1 (0.9%)	0 (0.0%)	0.179
Diarrhea	106 (95.6%)	4 (3.5%)	1 (0.9%)	0 (0.0%)	0 (0.0%)	0.203
Hearing loss	110 (97.3%)	3 (1.8%)	1 (0.9%)	0 (0.0%)	0 (0.0%)	0.523
Rash	97 (85.8%)	13 (11.5%)	3 (2.7%)	0 (0.0%)	0 (0.0%)	0.414
Alanine aminotransferase	80 (70.8%)	27 (23.9%)	6 (5.3%)	0 (0.0%)	0 (0.0%)	0.125
Aspartate aminotransferase	89 (78.8%)	18 (15.9%)	6 (5.3%)	0 (0.0%)	0 (0.0%)	0.039
The acute toxicities of radiotherapy						
Dermatitis	107 (94.7%)	3 (2.7%)	3 (2.7%)	0 (0.0%)	0 (0.0%)	0.114
Mucositis	84 (74.3%)	23 (20.4)	6 (5.3)	0 (0.0%)	0 (0.0%)	0.740
Dysphagia	99 (87.6%)	13 (11.5%)	1 (0.9%)	0 (0.0%)	0 (0.0%)	0.481
The late toxicities of radiotherapy						
Xerostomia	106 (93.8%)	6 (5.3%)	1 (0.9%)	0 (0.0%)	0 (0.0%)	0.773
Neck fibrosis	110 (97.3%)	3 (2.7%)	0 (0.0%)	0 (0.0%)	0 (0.0%)	0.592
Trismus	112 (99.1%)	1 (0.9%)	0 (0.0%)	0 (0.0%)	0 (0.0%)	0.330
Hearing impairment	103 (91.2%)	6 (5.3%)	4 (3.5%)	0 (0.0%)	0 (0.0%)	0.544
Cerebral injury	112 (99.1%)	1 (0.9%)	0 (0.0%)	0 (0.0%)	0 (0.0%)	0.330
Cranial nerve palsy	0 (0.0%)	0 (0.0%)	0 (0.0%)	0 (0.0%)	0 (0.0%)	1.000

The most common chemotherapy-related toxicity was related to haematological factors and vomiting (43.3% grade 1). Haematological factors included leukopenia (18.6% grade1), thrombocytopenia (21.2% grade1) and haemoglobin reduced leukopenia (42.5% grade1). Grade2 leukopenia was observed in 47.8% of patients and grade2 haemoglobin reductions occurred in 2.8% of patients. The worst toxicity type was grade 4 thrombocytopenia in one patient. Grade 3 leukopenia occurred in 15.0% of patients and grade 2 leukopenia occurred in two patients. Toxicity type and onset was not significantly difference between treatment group, including leukopenia (P = 0.189), thrombocytopenia (P = 0.794), haemoglobin reduction (P = 0.176), nausea and vomiting (P = 0.179), diarrhoea (P = 0.203), hearing loss (P = 523), rash (P = 0.414) and alanine aminotransferase (P = 0.125), Likewise, there were no difference between two arms in radiotherapy. However, differences were observed for aspartate aminotransferase (P = 0.039).

## Discussion

Previous researchers have performed multiple studies over many years. In 2006, Baujat stated that the addition of chemotherapy benefited an absolute 5-years survival rate of 6% 5-year^16^. Trial 0091^5^ laid the foundation for CCRT treatment in NPC. The local confinement rate had satisfactorily increased, but the distant metastasis rate still remained at 15–20%^17^. Besides the local disease control in locoregionally advanced NPC, distant metastasis is often the most important factor leading to poor prognosis. As a result, researchers developed a new therapeutic strategy called induction chemotherapy, followed by CCRT for locoregional NPC patients. A Network Meta-Analysis showed that CCRT plus induction chemotherapy improved distant metastatic control^18^. Recently, several prospective phase III trials proved that induction chemotherapy followed by CCRT could promote the survival rate for NPC (NCT00201396, NCT00705627, and NCT01872962). Hao Peng *et al*. evaluated the long-term therapeutic gain of induction chemotherapy as an effective treatment modality for patients in locoregionally advanced NPC^2^.

A phase II study suggested that the addition of docetaxel into the induction chemotherapy group of cisplatin and 5-FU, prolonged the treatment outcomes in head and neck carcinoma^19^. Furthermore, a randomised phase III trial (NCT00003888) published in The New England Journal of Medicine, reported that induction chemotherapy with cisplatin, 5-FU plus docetaxel (TPF), was comparable to the regimen of cisplatin and 5-FU (PF), but significantly improved PFS and OS in patients with locoregionally advanced head and neck cancers^20^. Taxane-containing induction chemotherapy improved the treatment outcomes equally in patients with locally advanced NPC compared with the non-taxane chemotherapy^6,21^.

Gemcitabine is a nucleoside analogue which is a broad anticancer agent^13^. Gemcitabine and cisplatin induction chemotherapy was superior in locoregionally advanced NPC^12,14,22^. Wang *et al*. indicated that the 4-year OS rate of GP-based induction chemotherapy before CCRT was 81.9%, with fewer severe haematological adverse events in locoregionally advanced NPC^14^. A retrospective study performed by Zhao *et al*. reported that GP regimens had a higher OS rate than the PF regimen, and demonstrated a trend towards better survival rates^22^. Our study aimed to compare the treatment outcomes between TPF and GP induction chemotherapy treatments followed by CCRT in locoregionally advanced NPC. No significant differences were found between the two regimens in OS, PFS, LRFS and DMFS.

The 3-year estimated OS of patients treated with TPF induction chemotherapy regimen was 87.9% and 87.4% with GP chemotherapy plus CCRT (Log-Rank P = 0.928). The 3-year PFS of the TPF group was 84.5%, while it was 83.5% for the GP regimen. However, there were no statistical differences between TPF and GP treatment strategies for OS (Log-Rank P = 0.928) or PFS (Log-Rank P = 0.551; Fig. 2B) in locoregionally advanced NPC. During the 3-year follow-up period, the TPF treatment appeared to have a small advantage in terms of OS and PFS over the GP regimen. Induction chemotherapy can promote the DMFS rate in locoregionally advanced NPC^18^. In our study, the DMFS between TPF and GP treatment was not statistically different (p = 0.892). However, the GP treatment showed a trend towards a better DMFS in comparison to the TPF regimen (Fig. 2D). N stage, nausea and vomiting, and ECOG performance were independent prognostic factors for DMFS in the univariate analysis. N3 stage was observed to be the main independent prognostic element for PFS and DMFS in the multivariate analysis. In the study, 16 of the patients were observed to have different degrees of skin rash following treatment, with 13 people suffering a first-degree skin rash, and 3 patients developing a second-degree skin rash. Development of a skin rash appeared to be an independent prognostic indicator in both the univariate and multivariate analysis.

Regarding adverse toxicity following induction chemotherapy, all the toxicity events observed were not statistically different between the TPF and GP regimen, except for increasing of aspartate aminotransferase (AST). Each adverse toxicity event was well- tolerated by the NPC patients receiving both treatment strategies, and the vast majority of side effects occurred in grade 1 or 2 categories, with only a few occurring in grade 3 or 4. Among them, leukopenia and nausea and vomiting were classified as grade 3 or 4, but they did not interrupt the administration of treatment. The TPF regimen had more grade 3 or 4 adverse events than the GP treatment. Taking leukocytes as an example, the probability for the appearance of grade 3 or 4 adverse reactions in the TPF group was 10.6%, while only 4.4% in GP regimen. The increase of aspartate aminotransferase (AST) was regarded as the only observed adverse reaction, which had any statistical significance between two groups. However, after following up and collecting the medical history of the patients, we found that most of those with high AST were suffering from chronic hepatitis B in the corresponding period, especially those categorised as grade 2 AST patients. As a result, chronic hepatitis B was a complex factor contributing to the increase of AST. AST as a clinical index reflects damage to liver function. For HBV (Hepatitis B Virus)-infected patients with cancer, anti-cancer therapy may lead to reactive HBV and damage liver function^23–25^. The high AST of the two treatment plans may contribute no significant difference.

Our retrospective study was limited by the small sample size and the possibility of patient selection bias with a short follow-up time. A future large scale randomised clinical trial is warranted to further the investigation with a longer-term follow-up.

Therefore, we conclude that there was no significant difference in treatment outcomes for the TPF or GP induction chemotherapy strategies, for the treatment of locoregionally advanced NPC. Furthermore, the adverse toxicities were similar and could be tolerated. However, the TPF group had a high proportion of grade 3 or 4 adverse reactions. In addition, the cost ratio of GP to TPF is lower. In clinical practice, alopecia occurred less frequently in patients treated with GP chemotherapy.

## Methods

### Patient characteristics

This study was a retrospective, matched case–control trial derived from an existing database. Patients with locally advanced NPC that were treated at the West China Hospital Cancer Center between May 2009 and Dec 2014 were enrolled in this study. Fifty-eight patients were enrolled in TPF induction chemotherapy regimen group, and Fifty-five patients completed GP regimen. Both groups received CCRT incorporating IMRT and cisplatin-based chemotherapy. Eligibility criteria for patients included a Karnofsky performance score above or equal to 70 points, and to have confirmed stage III–IV NPC with non-distant metastases.

### Induction chemotherapy and concurrent chemotherapy

The induction chemotherapy regimens were divided into two treatment groups with each administration cycle occurring every 3 weeks. In the first group, patients received 2–3 cycles of the TPF regimen intravenously (IV), consisting of docetaxel (60 mg/m² IV on day 1) cisplatin (75 mg/m² IV on day 1 or within 3 days), and 5-FU (600 mg/m² IV on days 1 to 5). This induction intervention occurred over approximately 6–9 weeks followed by a 3–4 week rest period before the initiation of the CCRT regimen. The second induction group consisted of patients receiving 2–3 cycles of the GP regimen via an IV infusion (gemcitabine 1000 mg/m² IV on day 1 and day 8, with cisplatin 75 mg/m² IV administered on day 1 or within 3 days), with each cycle delivered approximately every 3 weeks. Overall, the induction chemotherapy for both groups was given usually every 3 weeks for a total period of 6–9 weeks, followed by a 3–4 week rest period before the start of the CCRT. Therefore, CCRT was administered 12–13 weeks after initiation of induction chemotherapy, and CCRT was administered depending on physical tolerance.

### Concurrent radiotherapy

RT was initiated 3–4 weeks following completion of the induction chemotherapy regimens. IMRT was selected and the NCCN (National Comprehensive Cancer Network) guidelines for NPC RT were followed. The gross tumour volume (GTV) was determined by measuring the nasopharyngeal primary tumour area (GTVnx) by clinical and radiological examination, and by measuring the enlarged lymph nodes (GTVnd) by clinical touch and imaging techniques including nasopharyngeal and cervical enhanced magnetic resonance imaging (MRI) and enhanced computed tomography (CT). Multiple GTVnd could be detected according to the neck cervical lymph nodes. The high-risk clinical tumour volume (CTV1) included GTVnx and its surrounding subclinical lesions 5–10 mm out of range of the GTV. The low-risk clinical tumour volume (CTV2) always expanded from CTV1 and included the Skull base bone, a third of the sphenoid sinus, posterior ethmoid sinus, a third of the nasal cavity, pharynx, and retropharyngeal space. The planning target volume (PTV) was defined as the nasopharynx clinical tumour volume plus a 5-mm margin to encompass the CTV and to ensure that the actual dose administered reached the prescription dose. Depending on the differences in nasopharyngeal primary lesions, subclinical lesions, and the cervical lymph node drainage area, different doses of radiation were administered. A total prescribed dose of 66–74 Gy to the PTV was administered for the gross primary tumour site and positive lymph nodes. This consisted of 33–35 fractions with 1.8–2.0 Gy per fraction, administered daily from Monday to Friday for 6–7 weeks. This regimen helped to improve local tumour dosing and protected the normal, adjacent tissue.

### Procedures

Patients underwent TPF or GP induction chemotherapy regimens, followed by CCRT with single drug chemotherapy (cisplatin 80 mg/m^2^ IV on day 1 or within 3 days every 3 weeks) and IMRT. The main purpose of the present study was to investigate OS, which was defined as the specific period from the start of treatment to the cancer-related death. The secondary goals were to determine local recurrence-free survival (LRFS), defined as the period from the start of treatment to the first occurrence of nasopharyngeal or cervical lymph region recurrence; DMFS, defined as the period from the start of treatment to the first occurrence of metastasis; and the progressive-free survival (PFS), defined as the period from the start of treatment to the first occurrence of disease progression. Short-term responses of treatments were evaluated according to CT of radiotherapy localization and MRI of head and neck after IC and three months after CCRT. It’s based on the Response Evaluation Criteria in Solid Tumours (RECIST), which were divided into complete remission (CR), partial remission (PR), stable disease (SD), and progressive disease (PD). Radiation-related acute and chronic toxicities were based on the Radiation Morbidity Scoring Criteria of the RTOG (radiation therapy oncology group), and chemotherapy-related acute and chronic toxicities were based on the Common Terminology Criteria for Adverse Events (CTCAE) version 3.0. We carried out regular follow-up with patients, including head and neck examination, nasopharyngoscopy, nasopharyngeal MRI and neck MRI, Chest CT, abdomen ultrasound, haematology and biochemistry profiles. The date of the last follow-up was in June 2017.

### Statistical analysis

Treatment survival outcomes including, OS, PFS, LRFS, and DMFS were calculated using nonparametric statistics, namely Kaplan–Meier survival analysis and compared with log-rank and Wilcoxon tests. We used the log-rank test to compare survival curves. Univariate analyses were performed using Kaplan–Meier survival analysis and then selected into multivariate analysis to determine the possible prognostic risk factors that were associated with treatment efficiency. Chi-square and/ or Fisher’s exact tests were used to examine whether T stage, N stage, clinical stage and groups were independent based on the patients’ data. In all the statistical tests, P values less than 0.05 between each group were deemed to show a statistically significant difference. All analyses were performed by using by SPSS Statistics 19.0 for Windows (SPSS, Chicago, IL).

### Ethical approval and informed consent

Ethics approval of this study was approved by the Institutional Review Board of West China Hospital of Sichuan University, China. Without personal information of these patients, the institutional review board declared that the written consents of participants were not needed. Using anonymity protected all patients from leaking privacy.

## Data Availability

Data are available on request from the first author.
